# Abrupt current switching in graphene bilayer tunnel transistors enabled by van Hove singularities

**DOI:** 10.1038/srep24654

**Published:** 2016-04-21

**Authors:** Georgy Alymov, Vladimir Vyurkov, Victor Ryzhii, Dmitry Svintsov

**Affiliations:** 1 Department of Physical and Quantum Electronics, Moscow Institute of Physics and Technology, Dolgoprudny 141700, Russia; 2 Laboratory of Sub-micron Devices, Institute of Physics and Technology RAS, Moscow 117218, Russia; 3 Research Institute of Electrical Communication, Tohoku University, Sendai 980-8577, Japan

## Abstract

In a continuous search for the energy-efficient electronic switches, a great attention is focused on tunnel field-effect transistors (TFETs) demonstrating an abrupt dependence of the source-drain current on the gate voltage. Among all TFETs, those based on one-dimensional (1D) semiconductors exhibit the steepest current switching due to the singular density of states near the band edges, though the current in 1D structures is pretty low. In this paper, we propose a TFET based on 2D graphene bilayer which demonstrates a record steep subthreshold slope enabled by van Hove singularities in the density of states near the edges of conduction and valence bands. Our simulations show the accessibility of 3.5 × 10^4^ ON/OFF current ratio with 150 mV gate voltage swing, and a maximum subthreshold slope of (20 *μ*V/dec)^−1^ just above the threshold. The high ON-state current of 0.8 mA/*μ*m is enabled by a narrow (~0.3 eV) extrinsic band gap, while the smallness of the leakage current is due to an all-electrical doping of the source and drain contacts which suppresses the band tailing and trap-assisted tunneling.

The design of field-effect transistors (FETs) operating at sub-0.5 V supply voltage is one of the major challenges for nanoelectronics paving the way to resolve the problem of power dissipation in large integrated circuits. Tunnel FETs (TFETs) with interband tunneling are among the principal candidates to meet this demand^1^,^2^. The low-voltage switching in TFETs occurs due to a sharp dependence of the tunnel current on the conduction-valence band overlap in a gate-controlled *p*-*i* or *p*-*n* junction^3^; once there is no band overlap, there is no tunnel current. This fact, alongside with the smallness of thermionic leakage current and efficient modulation of the barrier transparency by the gate voltage, results in the subthreshold slope of the TFET characteristics surpassing the thermionic limit^4^,^5^,^6^,^7^,^8^ of (60 mV/dec)^−1^.

It is intuitive that the abrupt variations of electron and hole densities of states (DoS) near the band edges further enhance the switching efficiency of TFETs^9^. For a *d*-dimensional TFET channel, the DoS scales with energy *E* above the band edge as *E*^(*d*−2)/2^, while the dependence of current *J* on the gate voltage *V*_*G*_ above the threshold *V*_*T*_ is *J* ∝ [*V*_*G*_ − *V*_*T*_]^(*d*+1)/2^ 10,11. Apart from the density-of-states-enhanced switching, the TFETs with low-dimensional channels demonstrate an improved electrostatic control of the band alignment by the gate voltage^8^,^12^,^13^. Theoretically, the effects of DoS on current switching steepness are most pronounced in vertical TFETs based on the two-dimensional crystals^14^ and electron-hole bilayers in quantum-confined structures^15^,^16^. In such TFETs, the joint density of states is nonzero just at one certain value of gate voltage^17^ – which could lead to the abrupt-most current switching ever. In practice, the density-of-states effects on the subthreshold steepness are largely smeared. The reason for the smearing in vertical TFETs based on van der Waals heterostructures is the rotational misalignment of 2D layers in momentum space^18^. In common semiconductor structures it is believed that charged defects and dopants lead to pronounced band-tailing and emerging trap-assisted and band-tail tunneling leakage currents^19^,^20^,^21^.

In this paper, we theoretically demonstrate that graphene bilayer (GBL) represents an ideal platform for the low-voltage tunnel switches due to its peculiar ‘mexican hat’ band structure of GBL formed under transverse electric field^22^,^23^ and a van Hove singularity in the DoS right at the bottom of the band edges, as shown in Fig. 1A. The experimental evidence for this singularity were obtained already for graphene samples on disordered SiO_2_ substrates: the measurements of infrared absorption^24^, quantum capacitance^25^, and tunnel current in scanning probe microscope^26^ indicated the large density of states. The emergence of high-quality boron nitride substrates for graphene electronic devices started a new chapter in the experimental studies of low-energy spectrum of bilayer, and not only the van Hove singularities^27^,^28^ but even tinier features of carrier spectrum^29^ were clearly revealed. In our paper we show that the van Hove singularity results in a steep, linear dependence of the GBL TFET current on the gate voltage above the threshold, which was attributed previously just to the TFETs based on one-dimensional materials^5^,^8^,^30^,^31^,^32^.

The advantage of graphene bilayer TFETs over those based on 2d materials with parabolic bands in terms of switching steepness can be illustrated by Fig. 2B. As the conduction and valence bands in a GBL tunnel junction overlap, the electrons capable of tunneling are located on a ring in the momentum space. In contrast, the electrons capable of tunneling between simple parabolic bands are located in a small vicinity of an extreme point of the dispersion. Under optimal gate biasing conditions, the proposed TFET demonstrates the current switching over more than 4 orders of magnitude with 150 mV gate voltage swing only. At the same time, the ON-state current density as large as 0.8 mA/*μ*m is accessible due to the low extrinsic band gap of GBL (~0.3 eV) and large DoS far above the band edges.

Although a number of GBL transistors have been proposed^33^,^34^,^35^, including the TFETs^36^, our structure possesses a unique feature that allows one to exploit the density-of-states effect for tunneling. This feature is electrical doping of source and drain contacts instead of chemical one. This suppresses the band tailing induced by random dopants^37^ and minimizes the leakage currents through defect states^38^. Apart from reducing the leakage, this adds the possibility to electrically reconfigure the device between *n*- and *p*-types.

## Results

### Device structure

The advantages of graphene bilayer for the steep current switching can be fully realized in the structure of the TFET shown schematically in Fig. 2A. A heavily doped silicon substrate acts as a bottom gate used to create the transverse electric field and thus open and manipulate the band gap in GBL^39^. The oxidation of the substrate results in formation of SiO_2_ layer playing the role of back gate oxide and substrate for graphene bilayer. Alternatively, the SiO_2_ layer can be replaced with hexagonal boron nitride (hBN) possessing a small (~10^10^ cm^−2^) density of residual charged impurities^40^. A nanometre – thin layer of high-*κ* dielectric (e.g., zirconium oxide) covers the graphene channel, and the top metal gates are formed above. The side gates near the source and drain contacts induce large densities of holes and electrons, respectively, which also leads to the formation of an abrupt tunnel junction and energy barriers (see Fig. 2B) for the thermally activated electrons and holes contributing to the OFF-state leakage current.

The operation of a normally open TFET switched off by a negative top gate voltage is illustrated in the band diagram, Fig. 2B. Application of positive voltage to the bottom gate, *V*_*B*_ > 0, induces the band gap and provides an excess electron density in bilayer. The *p*^+^ doping of source emerges upon application of negative voltage *U*_*S*_ < 0 to the source doping gate. An additional increase in the barrier height for the holes injected from the drain is achieved by applying positive voltage *U*_*D*_ > 0 to the drain doping gate. It is instructive that application of high voltage to the doping gates does not result in increased power consumption as this voltage is not changed during the device operation. At zero top gate voltage, the valence band in *p*^+^–source overlaps with the conduction band in the *n*–type channel, which corresponds to the ON state (red band profiles in Fig. 2B). Upon application of negative voltage to the top gate, the transistor is switched off (dashed blue band profiles in Fig. 2B).

The optimization of the device dimensions aiming at the increase in the ON-state and reduction in the OFF-state currents is quite straightforward: both the effective thickness of the gate dielectric and the distance between the source doping gate and the control gate should be small. These distances are limited just by the possible gate leakage current (see below), we choose them to be *d*_*t*_ = 2 nm and *d*_*g*_ = 5 nm. The doping gate at the drain is used just to induce high barrier for thermally activated holes; the distance between this gate and the control gate should be large to reduce the transparency of tunnel junction at the drain and get rid of ambipolar leakage.

The fabrication of the device structure in Fig. 2A is technologically feasible with recent advances in the growth of graphene on hBN^41^. The most challenging operation is the formation of the gates at sub-10 nm distance, which is, however, achievable with the combination of self-assembled molecular and electron beam lithographic techniques^42^.

### Model of the interband tunneling enhanced by van Hove singularities

**Figure 1:**
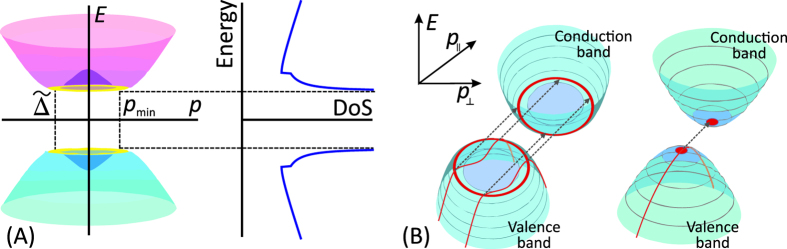
(**A**) Electron spectrum *E*(*p*) in graphene bilayer under transverse electric field and the energy dependence of its DoS. The “Mexican hat” feature in the dispersion law leads to the square-root singularities in the DoS near the band edges. Panel (**B**) highlights with red the electron states involved in the interband tunneling at small band overlap in graphene bilayer (left) and in a semiconductor with parabolic bands (right). The phase space for tunneling in graphene bilayer represents a ring, while in a parabolic-band semiconductor it is a point. Dashed lines indicate the tunneling transitions, red lines indicate the trajectories of the tunneling electrons in the valence band.

**Figure 2:**
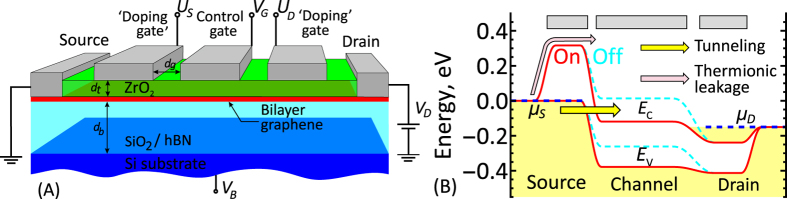
(**A**) Layout of the proposed graphene bilayer TFET with electrically defined source and drain regions (**B**) Band diagram of graphene bilayer TFET for the optimal biasing conditions: *V*_*B*_ > 0, *U*_*S*_ < 0, *U*_*D*_ > 0. At zero top gate bias, *V*_*G*_ = 0, the TFET is switched on, while at *V*_*G*_ < 0 it is switched off.

Our modeling of graphene bilayer TFET relies on a self-consistent determination of the carrier density and band structure^22^ followed by the calculation of tunnel current under assumption of ballistic transport (see supplementary material, sections I–III). However, the principal dependence of the tunnel current on the gate voltage can be derived in a very simple fashion. The current is proportional to the number of electrons capable of tunneling between the valence band of source and the conduction band of channel. Once these band overlap by *dE* in the energy scale, the electrons available for tunneling in graphene bilayer occupy a ring in the momentum space (Fig. 1B, left panel). Their number is proportional to *p*_min_*dE*, where *p*_min_ is the momentum corresponding to the bottom of the ‘Mexican hat’. One thus concludes that the tunnel current is a linear function of the band overlap which, in turn, is a linear function of the gate voltage. This contrasts with the 2d materials having parabolic bands where the number of electrons available for tunneling is proportional to 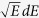 (Fig. 1B, right panel). As a result, the current in TFETs based on these materials is proportional to the gate voltage raised to the power 3/2.

A rigorous expression for the tunnel current density involves an integral of the single-particle velocity *v*_||_ = *dE*/*dp*_||_ timed by the barrier transparency 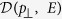 and the difference of occupation functions in the valence and conduction bands *f*_*v*_(*E*) − *f*_*c*_(*E*) over the momentum space *d*^2^**p** = 2*dp*_⊥_*dp*_||_^10^:


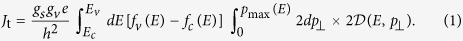


Here, *g*_*s *_*g*_*v*_ = 4 is the spin-valley degeneracy factor in graphene, *p*_max_(*E*) is the maximum transverse momentum of electron at a given energy *E*, *p*_max_(*E*) = min{*p*_*c*_(*E*), *p*_*v*_(*E*)}, where *p*_*c*_(*E*) and *p*_*v*_(*E*) are the inverse functions to the electron dispersion in the conduction and valence bands. The limits of integration over energy are the conduction band edge in the channel, *E*_*c*_, and the valence band edge in the source, *E*_*v*_. The factor of two before the quasi-classical barrier transparency comes from the presence of two turning points with zero group velocity in the GBL dispersion at which an electron attempts to tunnel.

The effect of ‘Mexican hat’ on the current switching steepness can be traced analytically from Eq. 1 by assuming that the conduction band states are empty, valence band states are occupied, and the barrier transparency 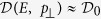 weakly depends on the energy and transverse momentum. At small band overlap, the momenta of the tunneling electrons in graphene bilayer are close to *p*_min_ (Fig. 1B, left panel), which results in


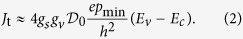


This linear dependence is in agreement with the above qualitative considerations. Previously, such a dependence of the tunnel current on the band overlap was attributed just to the 1D semiconductor structures which proved to be among the best candidates for the TFETs^5^,^7^,^43^.

An additional increase in the graphene bilayer TFET subthreshold steepness occurs due to the dependence of the transparency 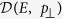 on the junction field and, hence, gate voltage. The transparency is evaluated by integrating the imaginary part of the electron momentum inside the band gap, which results in (see Supporting information, section III)


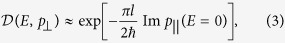


where Im *p*_||_(*E* = 0) is the imaginary part of electron momentum evaluated at the midgap, and *l* is the length of the classically forbidden region (tunneling path length). The latter is given by 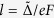 for *p*_⊥_ < *p*_min_, and *l* = 2*E*(*p*_⊥_)/*eF* for *p*_⊥_ > *p*_min_, where *F* is the electric field at the junction found from the solution of Poisson’s equation, and 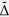 is the band gap in the GBL. The thermionic leakage currents were evaluated with equations similar to (1) by setting the unity transmission probability and constraining the energy integral to the particles with the energies above the barrier.

### Characteristics of the graphene bilayer TFET

**Figure 3:**
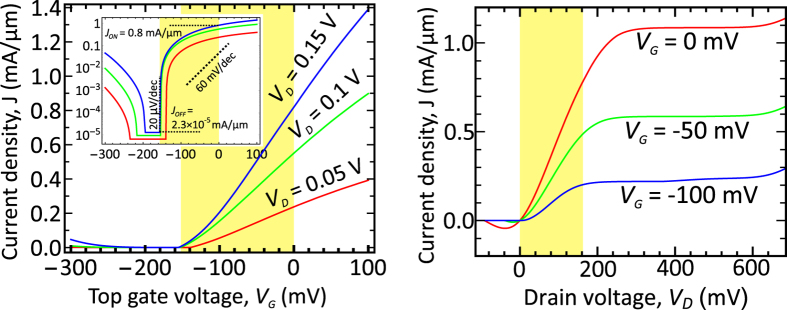
Calculated room-temperature gate transfer (left) and current-voltage (right) characteristics of graphene bilayer TFET at fixed bias voltages at auxiliary gates: *V*_*B*_ = 3.3 V, *U*_*S*_ = −0.6 V, *U*_*D*_ = 0.25 V. Top gate dielectric is 2 nm ZrO_2_, *κ* = 25, back gate dielectric is 10 nm SiO_2_, spacing between the source doping and control gates *d*_*g*_ = 5 nm, spacing between drain doping and control gates is 10 nm. The regions highlighted in yellow correspond to the drive voltage swing of 150 mV, in which sufficient ON/OFF ratio and high ON-state current are achieved. Inset: gate transfer characteristic in the log scale.

The calculated room-temperature *J*(*V*_*G*_)–characteristics of graphene bilayer TFET at different drain bias *V*_*D*_ are shown in Fig. 3A. The current density just above the threshold voltage is a linear function of *V*_*G*_, in agreement with the simple density-of-states arguments and Eq. (2). With increasing the top gate voltage, the slope of *J*(*V*_*G*_)-curve slightly increases due to the sensitivity of the tunnel barrier transparency to the junction field. The subthreshold slope at *V*_*G*_ = *V*_*th*_ reaches (20 *μ*V/dec)^−1^ and is limited by the small thermionic current 
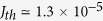
 mA/*μ*m and the gate leakage current 
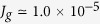
 mA/*μ*m. About 100 mV below the threshold, the ambipolar leakage at the drain tunnel junction becomes pronounced; this can be, however, minimized by placing the drain doping gate at large distance from the control gate.

The drain characteristics of graphene bilayer TFET shown in Fig. 3B demonstrate a pronounced current saturation typically absent in single graphene layer FETs. This saturation is due to the limited energy range in which the tunneling injection is possible. At very high drain bias ~600 mV, the barrier for thermally activated holes at drain junction is sufficiently lowered, which leads to the further increase in current. At negative drain bias, the transistor current can be viewed as that of *p*^+^ − *n*^+^ tunnel diode between source and channel. The emerging negative differential resistance is due to the dependence of the band edges in the channel on the amount of injected carriers: at high electron density, the bands in the channel are lifted upwards, which reduces the source-channel band overlap and switches the tunneling off.

For low-power applications, the maximization of the highest subthreshold slope is not as important as minimization of the supply voltage *V*_*S*_ required to switch the transistor between the ON- and OFF-states. Considering the current at *V*_*G*_ = 0 V as the ON-state current (*J*_*ON*_ = 0.8 mA/*μ*m in Fig. 3B at *V*_*D*_ = 0.15 V), and the leakage current as the OFF-state current (*J*_*ON*_/*J*_*OFF*_ = 3.5 × 10^4^), we have obtained *V*_*S*_ = 150 mV. In a conventional MOSFET, the gate voltage swing *V*_*S*_ ≥ 285 mV is required to achieve the same current switching ratio. The average subthreshold slope of our TFET over 4.5 decades of current is 33 (mV/dec)^−1^. With this characteristic, it outperforms all sub-thermal tunnel switches^2^ based on silicon^4^, germanium^6^, III-V hetero junctions^5^, and carbon nanotubes^7^ reported to date. Only recently a vertical TFET based on MoS_2_/germanium junction with a similar value of the average subthrehold slope was demonstrated^44^, however, its ON-state current density of 1 *μ*A/*μ*m leaves much to be desired.

The aggregate quality of the TFET, accounting for both average subthreshold slope and current density, can be characterized by an *I*_60_–figure of merit^45^ which is the current density at the point where the subthreshold slope equals (60 mV/dec)^−1^. While the best *I*_60_ reported to date equals 6 nA/*μ*m (InAs nanowire/Si heterojunction TFET^5^), in our TFET structure *I*_60_ = 150 *μ*A/*μ*m.

**Figure 4:**
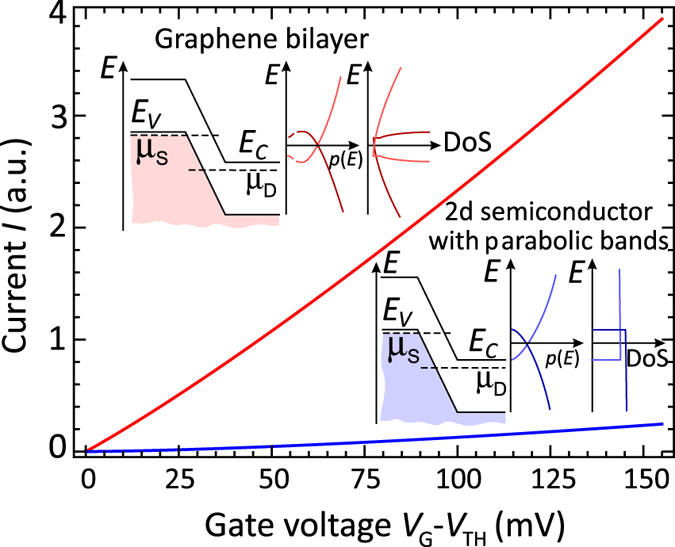
Comparison of the gate transfer characteristics of GBL TFET and a TFET based on an equivalent 2D semiconductor with the same barrier transparency *D*_0_, but with different (parabolic) band structure. Numerical values of the effective masses are taken for bulk InAs. The insets show the band diagrams overlaid with electron-hole spectra and the energy dependence of DoS.

The unique characteristics of GBL TFET surpassing the existing TFETs are enabled by the three factors. First of all, it is the small extrinsic band gap (for doping gate voltages used in Fig. 3, 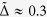 eV) that guarantees elevated tunneling probability (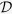
~0.1) and large current density. It is remarkable that there exists a lower limit of the interband transparency in GBL due to the saturation of the band gap 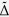 at high transverse field, 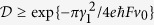, where *γ*_1_ ≈ 0.4 eV is the interlayer hopping integral and *v*_0_ ≈ 10^6^ m/s is the band velocity. Such transparency is sufficient to reach appreciable ON/OFF ratio, and it still enables pronounced ON-state current. At the same time, most semiconducting monolayers have large intrinsic band gaps (1.9 eV for MoS_2_, 1.3 eV for WS_2_, etc.), while in the 2D structures based on III–V materials being narrow-gap in the bulk, the gap value increases significantly due to the quantum confinement^46^. Secondly, the singular DoS near the band edges allows an abrupt switching of tunnel current. Even if there existed a parabolic-band 2D material with the same band gap and the same barrier transparency in the TFET structure, its current density would be given by (see supplemental material, section IV)


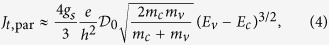


where *m*_*c*_ and *m*_*v*_ are the conduction and valence band effective masses and, similar to the derivation of Eq. (2), we have assumed the barrier transparency 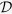
_0_ to be energy- and momentum independent. Last but not least, it is large density of states in GBL growing linearly at *high* energies that contributes to the high ON-state current. The numerical comparison of current density in graphene bilayer and its equivalent parabolic band counterpart is presented in Fig. 4 for the effective mass values typical for narrow-gap III–V semiconductors (*m*_*c*_ = 0.024*m*_0_, *m*_*v*_ = 0.026*m*_0_ for InAs). At 150 mV gate voltage above the threshold, the current density in graphene bilayer exceeds 15 times that in a parabolic-band material. The factor of two is due to the valley degeneracy absent in III-V’s, another factor of two is due to the tunneling at two turning points of the ‘Mexican hat’ dispersion, and the remainder of 3.5 is due to the finiteness of electron momentum at the edge of the ‘Mexican hat’.

### Gate leakage and band tailing: the insulator selection rules

The steep switching of the tunnel current by the gate voltage can be masked by the leakage to the gates, band-tail and trap-assisted tunneling^19^,^20^,^21^. The latter factors might have masked the onset of the interband current in the recent measurements of graphene bilayer tunnel junctions^38^,^47^. A careful selection of the gate dielectrics providing high interface quality is required to minimize these effects.

**Figure 5:**
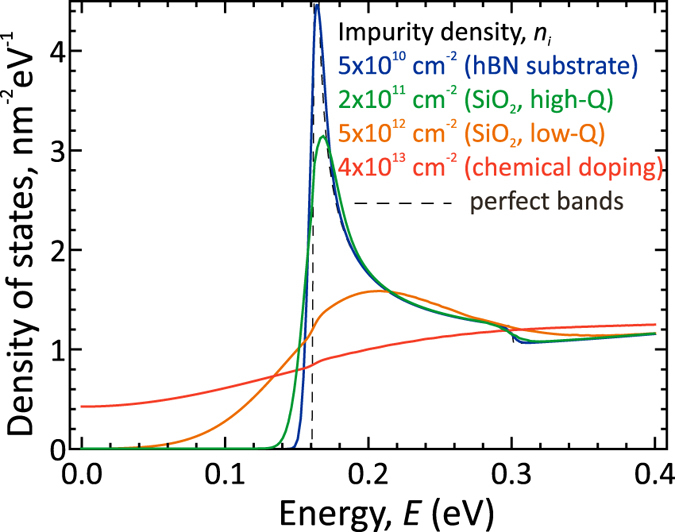
Calculated energy dependencies of the DoS in the conduction band of graphene bilayer at different densities of charged impurities (corresponding to the substrates of different quality). The electron density is held fixed at 4 × 10^13^ cm^−2^, the nominal energy gap is 0.3 eV.

The main reason for the band tailing comes from the fluctuations of electric potential produced by the random charged defects or dopants^37^. This effect is most pronounced in the TFETs with source and drain intentionally doped chemically. In the TFETs with electrically doped contacts, only residual charged impurities inevitably present in the substrate contribute to the band tailing. To provide a quantitative view on the band tailing in graphene bilayer on different substrates, we have evaluated the quasi-classical DoS *ρ*(*E*) in the presence of fluctuating potential by integrating the singular ‘bare’ DoS *ρ*_0_ over the probabilities of voltage fluctuations^37^


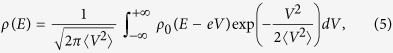


where 〈*V*^2^〉 is the root-mean-square amplitude of the voltage fluctuations proportional to the impurity density *n*_*i*_. The calculated energy dependencies of the ‘smeared’ DoS are shown in Fig. 5. For the parameters of chemical doping used in the pioneering proposal of the GBL TFET^36^, *n*_*i*_ = 4 × 10^13^ cm^−2^, the conduction and valence bands almost merge together, which would result in a poor OFF-state, nothing to say about high switching steepness. A slight peak in the DoS near the band bottom becomes noticeable already at impurity density of 5 × 10^12^ cm^−2^ which corresponds to the low-quality graphene on SiO_2_ substrates. In graphene samples on a high-quality SiO_2_^48^, the smearing of the band edge is order of 10 meV. The ultimate band abruptness of ~5 meV can be achieved in graphene samples encapsulated in boron nitride, providing the residual impurity density of ~5 × 10^10^ cm^−2^ 40. At this limit, the fluctuation-induced smearing of the bands becomes negligible, and the behavior of the DoS near the bottom of the “Mexican hat” is governed by the trigonal warping distortions of electron spectrum due to the next-nearest neighbor interactions^22^. Using the exact spectrum of GBL with trigonal warping, we estimate the energy scale where the trigonal warping is relevant as *δε* ≈ 20 meV. Already for relatively small gate voltages, *V*_*G*_ − *V*_*th*_ > *δε*/*e*, these corrections are irrelevant and the linearity of the *J*(*V*_*G*_)–characteristic holds.

The gate leakage may also limit the minimum achievable OFF-state current, while at the same time small effective gate oxide thickness is required to efficiently control the band structure in the channel by the gate voltage. Among the common high-*κ* materials, zirconium oxide (*κ* ≈ 25) looks as an optimal solution for the GBL TEFT due to the large band offset with respect to graphene (*U*_*b*_ = 2.9 eV^49^) and elevated tunneling mass *m*_*t*_ ≈ 0.3*m*_0_^50^. We have evaluated the leakage current from graphene with electron (hole) density of *n*_*e*(*h*)_ into the metal gate to be (see Supporting information, section VI)


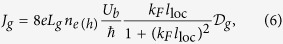


where 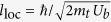 is the electron localization length in the direction perpendicular to the graphene bilayer, *k*_*F*_ is the Fermi wave vector in the metal, 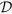
_*g*_ is the transparency of the barrier separating GBL and the gate, and *L*_*G*_ is the gate length. Under the biasing conditions of Fig. 3, the gate leakage current is estimated to be *J*_*t*_ = 1.0 × 10^−5^ mA/*μ*m which is below the thermionic leakage level (in this estimate, we have taken *L*_*g*_ = 20 nm and *k*_*F*_ = 2 Å^−1^).

## Discussion

We have proposed and substantiated the operation of a graphene bilayer TFET exploiting the van Hove singularities in the density of states near the band edges. The presence of these singularities leads to the increased steepness of the gate characteristics and to the high ON-state current as well. The subthreshold slope of *J*(*V*_*G*_) curve in the proposed FET reaches the maximum of (20 *μ*V/dec)^−1^, while only 150 mV gate voltage swing is required to change the current density from *J*_*ON*_ ≈ 1 mA/*μ*m down to *J*_*OFF*_ ≈ 2 × 10^−5^ mA/*μ*m. As a matter of fact, the effects of singular DoS on the interband tunneling are possible just in the TFET structures with an all-electrical doping, where the effects of band tailing and trap-assisted tunneling are minimized.

Such steep switching in the lateral TFETs based on 2d materials is possible if only the van Hove singularities are present both at the top of the valence and the bottom of the conduction band. This property is unique to the graphene bilayer and is absent in other 2d materials (e.g., those based on III–V compounds), where a ‘Mexican hat’ structure is formed in one of the bands due to the spin-orbit coupling^51^. We can thus conclude that graphene bilayer is an only two-dimensional material where the switching of interband tunnel current is as steep as in one-dimensional semiconductors, whereas the large on-state current is inherited from the single layer graphene.

**Figure 6:**
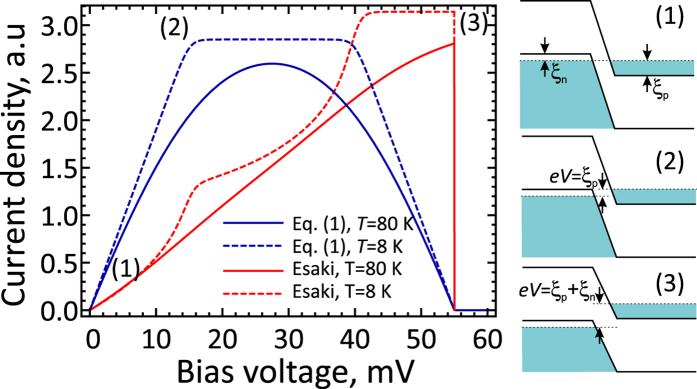
Characteristics of graphene bilayer *p*^+^ − *n*^+^ tunnel diodes calculated with our model, Eq. 1, blue lines, and with Esaki model^52^, red line. The temperature of 80 K corresponds to the experimental data obtained in ref. 38, *ξ*_*n*_ = 40 meV, *ξ*_*p*_ = 15 meV. The band diagrams correspond to the characteristic points (1), (2), and (3) of the *I*(*V*)-curve. In Esaki model, the current is proportional to the product of states densities and the difference of occupation functions integrated over the band overlap. The current calculated with Esaki model reaches its maximum as the van Hove singularities in the conduction and valence bands overlap, and then drops abruptly to zero. Such highly asymmetric peaks in tunnel current have never been observed in experiment^38^, which speaks in favor of our tunneling model.

At present, it is challenging to quantitatively compare the results our model to the experimental data, which are limited to the characteristics of graphene bilayer Esaki-type *p*^+^ − *n*^+^ tunnel junctions^38^. Our theory predicts a linear growth of diode current at small forward voltages, and a linear decrease in current at voltages 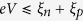, where *ξ*_*n*_ and *ξ*_*p*_ are the electron and hole Fermi energies in the *n*^+^ and *p*^+^-doped regions (see Fig. 6, blue lines). This behavior qualitatively agrees with the experimentally observed *I*(*V*)-curves. It is worth noting that a widely used phenomenological model of tunneling proposed by Esaki^52^, where the current is proportional to the integral of states’ densities in conduction and valence bands timed by the difference of occupation functions, predicts a different *I*(*V*)-curve. In such model, the current reaches its maximum at *eV* = *ξ*_*n*_ + *ξ*_*p*_ and then drops abruptly (Fig. 6, red lines). Such *I*(*V*) curves are not observed in the experiments, which speaks in favor of our rigorous model of tunneling.

## Methods

The modeling of GBL TFET is based on the self-consistent determination of carrier density and band structure under fixed gate voltages^22^ followed by the calculation of tunnel current with Eq. (1). The necessity for self-consistent calculation is dictated by the dependence of the energy gap on the electric field between graphene layers comprising the GBL; the field, in turn, depends on the induced carrier density which is sensitive to the band structure. The distribution of electric field at the tunneling junction required for the evaluation of the barrier transparency is calculated with the conformal mapping technique. The numerical model is described in detail in Supporting information, sections I-II. In section III of the Supporting information, an approximate analytic model of GBL TFET is presented.

The effect of charged impurities present in the substrate on the singular density of states in graphene bilayer is evaluated with Kane’s quasi-classical model of band tails^37^. The revision of the model for the two-dimensional GBL is presented in Supporting information, section V.

The gate leakage current is estimated with a quantum-mechanical model of graphene bilayer tunnel coupled to the continuum of delocalized states in the metal gate. The model is presented in Supporting information, section VI.

## Additional Information

**How to cite this article**: Alymov, G. *et al.* Abrupt current switching in graphene bilayer tunnel transistors enabled by van Hove singularities. *Sci. Rep.* **6**, 24654; doi: 10.1038/srep24654 (2016).

## Supplementary Material

Supplementary Information
