# Supplementary material for: Abrupt current switching in graphene bilayer tunnel transistors enabled by van Hove singularities
Source: Sci Rep. 2016 Apr 21;6:24654. doi: 10.1038/srep24654 (PMC4838945; doi:10.1038/srep24654)
Supplement: Supplementary Information [file srep24654-s1.pdf]

Supporting information to "Abrupt current switching in graphene bilayer tunnel transistors enabled by van Hove singularities"

Georgy Alymov[1,2], Vladimir Vyurkov[1,2], Victor Ryzhii[3], and Dmitry Svintsov[1,2,*]

[1]Department of Physical and Quantum Electronics, Moscow Institute of Physics and Technology, Dolgoprudny 141700, Russia
[2]Laboratory of Sub-micron Devices, Institute of Physics and Technology RAS, Moscow 117218, Russia
[3]Research Institute of Electrical Communication, Tohoku University, Sendai 980-8577, Japan
[*]svintcov.da@mipt.ru

ABSTRACT

In this document, we provide the details of graphene bilayer tunnel transistor modeling. We describe in detail the method for the self-consistent determination of band structure and carrier density in gated bilayer. We evaluate the distribution of electric potential at the tunnel junction, the respective interband transparency, and the tunnel current. The approximate analytical expression for the tunneling and thermionic currents are provided. Using the developed formalism for the calculation of tunnel current, we compare the characteristics of graphene bilayer TFET and a TFET based on a two-dimensional semiconductor with parabolic bands. Finally, we describe our method to estimate the band tails in disordered bilayer and the gate leakage current.

Self-consistent evaluation of band gap and carrier density in gated bilayer

The intrinsic graphene bilayer (GBL) is a gapless semiconductor with symmetric electron-hole dispersion being parabolic near the band edges. However, the application of transverse electric field $F_\perp$ leads to the potential energy difference $\Delta = eF_\perp d$ between the layers comprising GBL ($d = 3.35$ A is the interlayer separation in GBL) and opening of the band gap. As the transverse electric field depends on the carrier density, which, in turn, depends on the band structure, a self-consistent procedure for the determination of those quantities in gated GBL is required.

In this section, we describe the procedure used to evaluate the carrier density, bandgap, and electric potential in GBL at given potentials of top and bottom gates (V_t and V_b). The tight-binding Hamiltonian of graphene bilayer

$$\hat{H}(\mathbf{p}) = \begin{pmatrix} \frac{\Delta}{2} & v_0(p_x - ip_y) & \gamma_1 & 0 \\ v_0(p_x + ip_y) & \frac{\Delta}{2} & 0 & 0 \\ \gamma_1 & 0 & -\frac{\Delta}{2} & v_0(p_x + ip_y) \\ 0 & 0 & v_0(p_x - ip_y) & -\frac{\Delta}{2} \end{pmatrix} \quad (1)$$

yields the following energy bands depicted in Fig. 1:[1]

$$E_{1,4}(\mathbf{p}) = \pm\sqrt{\frac{\gamma_1^2}{2} + \frac{\Delta^2}{4} + v_0^2 p^2 + \sqrt{\frac{\gamma_1^4}{4} + v_0^2 p^2 \left(\gamma_1^2 + \Delta^2\right)}}, \quad (2)$$

$$E_{2,3}(\mathbf{p}) = \pm\sqrt{\frac{\gamma_1^2}{2} + \frac{\Delta^2}{4} + v_0^2 p^2 - \sqrt{\frac{\gamma_1^4}{4} + v_0^2 p^2 \left(\gamma_1^2 + \Delta^2\right)}}, \quad (3)$$

with the corresponding four–component eigen wave functions (non-normalized):

$$\Psi_i(\mathbf{p}) = \begin{pmatrix} \Psi_i^{A_{top}}(\mathbf{p}) \\ \Psi_i^{B_{top}}(\mathbf{p}) \\ \Psi_i^{A_{bottom}}(\mathbf{p}) \\ \Psi_i^{B_{bottom}}(\mathbf{p}) \end{pmatrix} = \begin{pmatrix} \left[\frac{\Delta}{2} - E_i(\mathbf{p})\right]\left\{\left[\frac{\Delta}{2} + E_i(\mathbf{p})\right]^2 - v_0^2 p^2\right\} \\ -v_0(p_x + ip_y)\left\{\left[\frac{\Delta}{2} + E_i(\mathbf{p})\right]^2 - v^2 p^2\right\} \\ \gamma_1\left[\frac{\Delta^2}{4} - E_i^2(\mathbf{p})\right] \\ \gamma_1 v_0(p_x - ip_y)\left[\frac{\Delta}{2} - E_i(\mathbf{p})\right] \end{pmatrix}. \quad (4)$$

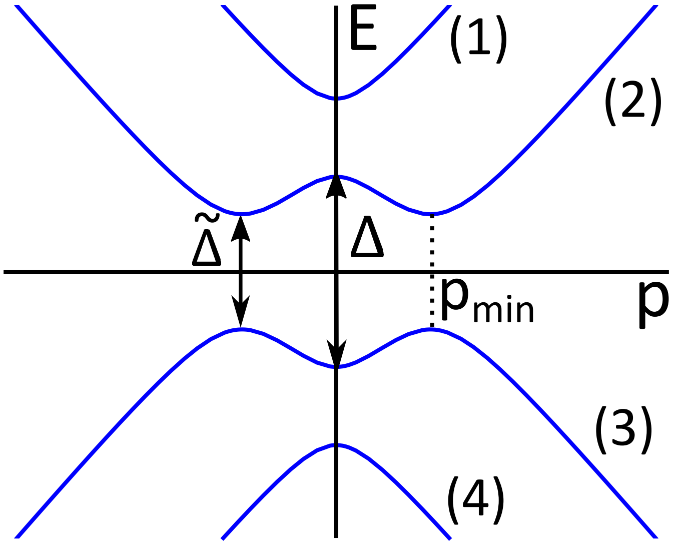

Figure 1. Band structure of graphene bilayer in tight binding approximation. Δ is the interlayer asymmetry parameter, $\tilde{\Delta} = \frac{\Delta\gamma_1}{\sqrt{\Delta^2+\gamma_1^2}}$ is the bandgap, and $p_{min} = \frac{\sqrt{\Delta^2+\tilde{\Delta}^2}}{2v}$ is the electron momentum at the band edges

The components of wave function determine the amplitudes of finding an electron on the atoms of type "A" and "B" of the unit cell on the top and bottom layers in the i-th band. In Eqs. (1) – (4), $\mathbf{p}$ is the electron quasimomentum, $\gamma_1 \approx 0.4$ eV is the interlayer hopping integral, and $v \approx 10^6$ m/s is the Fermi velocity in graphene. Given the eigenfunctions, the probabilities of finding an electron of the i-th energy band on the top and the bottom graphene layer can be calculated as follows:

$$w_i^t(\mathbf{p}) = \frac{\left|\Psi_i^{A_{top}}(\mathbf{p})\right|^2 + \left|\Psi_i^{B_{top}}(\mathbf{p})\right|^2}{\|\Psi_i(\mathbf{p})\|^2}, \quad (5)$$

$$w_i^b(\mathbf{p}) = \frac{\left|\Psi_i^{A_{bottom}}(\mathbf{p})\right|^2 + \left|\Psi_i^{B_{bottom}}(\mathbf{p})\right|^2}{\|\Psi_i(\mathbf{p})\|^2}; \quad (6)$$

here $||...||$ stands for the norm of the vector.

The net charge densities at top and bottom graphene layers are obtained by summing the probabilities (5) timed by the distribution functions over the bands and momenta:

$$\rho_t = eg\left\{\sum_{\mathbf{p},i=3,4} w_i^t(\mathbf{p})\left[1 - f(E_i(\mathbf{p}),\mu)\right] - \sum_{\mathbf{p},i=1,2} w_i^t(\mathbf{p}) f(E_i(\mathbf{p}),\mu)\right\}, \quad (7)$$

$$\rho_b = eg\left\{\sum_{\mathbf{p},i=3,4} w_i^b(\mathbf{p})\left[1 - f(E_i(\mathbf{p}),\mu)\right] - \sum_{\mathbf{p},i=1,2} w_i^b(\mathbf{p}) f(E_i(\mathbf{p}),\mu)\right\}. \quad (8)$$

Here, $g = 4$ is the spin-valley degeneracy factor, $f(E,\mu) = [e^{\frac{E-\mu}{kT}} + 1]^{-1}$ is the Fermi-Dirac distribution function, μ is the chemical potential reckoned from the midgap, k is the Boltzmann constant, and T is the absolute temperature.

We further consider graphene bilayer placed between the top and bottom gates separated by gate dielectrics of thicknesses d_t and d_b and dielectric constants ε_t and ε_b, respectively (see Fig. 2).

With the aid of Gauss law, the potential energy difference between layers, $\Delta = e(\varphi_b - \varphi_t)$, is readily expressed via the gate potentials V_t and V_b:

$$\Delta = ed\frac{V_b - V_t + \frac{\rho_b d_b}{\varepsilon_0\varepsilon_b} - \frac{\rho_t d_t}{\varepsilon_0\varepsilon_t}}{d + d_t/\varepsilon_t + d_b/\varepsilon_b}, \quad (9)$$

where ε_0 is the vacuum permittivity. Assuming that far from gates graphene is undoped ($\mu = 0$), and the source contact is

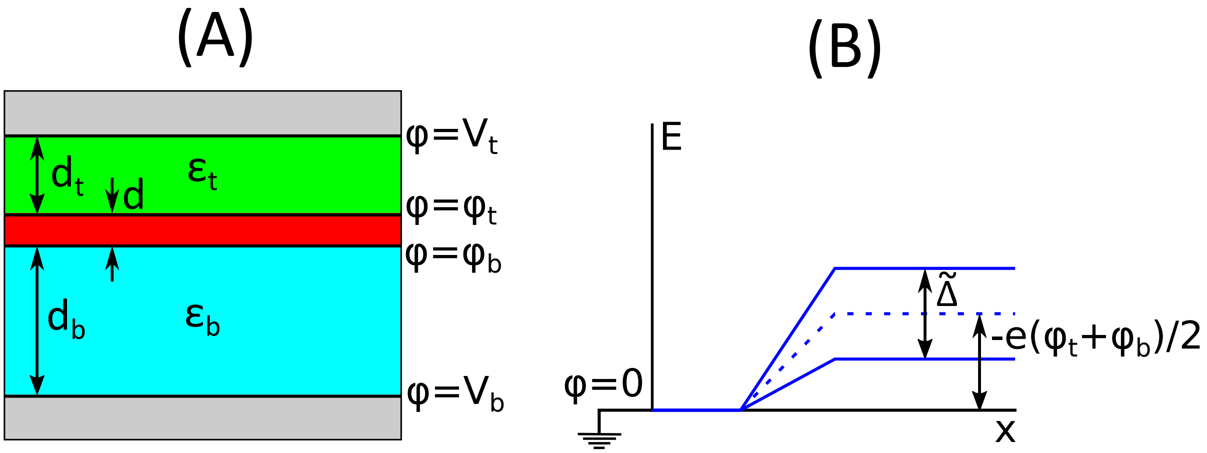

Figure 2. (A) Illustration of the electrostatic calculations. Electric field produced by top and bottom gates with potentials V_t and V_b induces potentials φ_t and φ_b at top and bottom layers of GBL. Panel (B) shows the band diagram in the source region. Near the grounded source contact there is no bandgap and GBL has zero potential, while under the "doping" gate GBL exhibits a bandgap $\tilde{\Delta}$ and has potential $(\varphi_t+\varphi_b)/2$, which leads to a corresponding midgap shift

grounded, we obtain an expression for the chemical potential in the source region:

$$\mu = e\frac{\varphi_t+\varphi_b}{2} = e\frac{\left(V_t+\frac{\rho_t d_t}{\varepsilon_0\varepsilon_t}\right)\left(\frac{d}{2}+\frac{d_b}{\varepsilon_b}\right)+\left(V_b+\frac{\rho_b d_b}{\varepsilon_0\varepsilon_b}\right)\left(\frac{d}{2}+\frac{d_t}{\varepsilon_t}\right)}{d+d_t/\varepsilon_t+d_b/\varepsilon_b}. \tag{10}$$

Equations (2)–(10) form a system of equations used for a self-consistent evaluation of Δ and μ in the source region. The system, which can be written in the symbolic form

$$\begin{pmatrix}\Delta\\ \mu\end{pmatrix} = \mathbf{F}(\Delta,\mu), \tag{11}$$

was solved numerically using an iterative method similar to the nonlinear successive over relaxation:[2]

$$\begin{pmatrix}\Delta^{i+1}\\ \mu^{i+1}\end{pmatrix} = (1-\lambda)\begin{pmatrix}\Delta^{i}\\ \mu^{i}\end{pmatrix} + \lambda\mathbf{F}(\Delta^i,\mu^i), \tag{12}$$

where i is the iteration number, and λ is the relaxation parameter adjusted to achieve convergence. The same approach was applied for the evaluation of Δ and μ in the drain region (with an obvious shift of electric potentials by the drain voltage). Considering the GBL band structure under the control (middle) gate, we assumed that the conduction band electrons are in equilibrium with the drain contact, while the holes are in equilibrium with the source contact. This approach is justified as far as the tunneling probability is small and the electrons injected from the valence band do not distort the potential distribution. To this end, two different chemical potentials for electrons and holes were used in Eqs. (7), (8) when calculating the charge density under the control gate.

Distribution of electric potential, barrier transparency, and current

The calculation of the tunnel current requires the knowledge of the barrier transparency which, in turn, depends on the distribution of electric potential in the tunneling region. Various studies of the tunnel diodes confirm that it is the maximum field in the junction region that governs the barrier transparency.[3–5] Thus, for an estimate of the tunnel current, the full knowledge of potential distribution is not necessary.

To obtain the junction field, we have solved the Laplace equation in the region between two gate electrodes: the "doping gate" above the source (held at potential U_S) and the top control gate above the channel (held at potential V_G) treating them as two keen metal plates.[6] The lateral distance between the gates is denoted by d_g. The effect of the bottom gate on the junction field is weak provided $d_b \gg d_t$. The difference between dielectric constants of top and bottom dielectric layers, $\varepsilon_t \neq \varepsilon_b$, can be approximately taken into account by replacing the top gate oxide thickness with effective oxide thickness, $d_{eff} = d_t\varepsilon_b/\varepsilon_t$. The distribution of electric potential in the geometry considered is obtained with conformal mapping technique, and the result is

$$\varphi(x,y) = \frac{U_S+V_G}{2} + \frac{2}{\pi}\frac{V_G-U_S}{2}\mathrm{Re}\left[\arcsin\left(\frac{x+iy}{d_g/2}\right)\right] \tag{13}$$

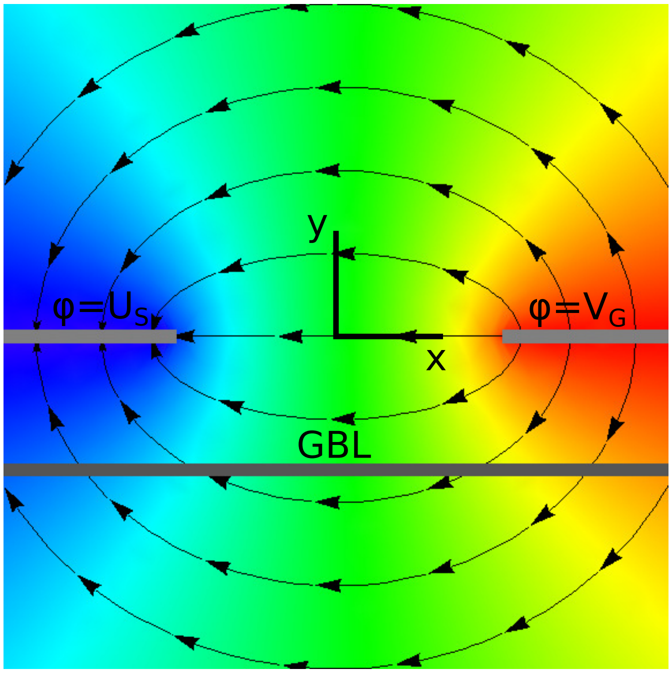

Figure 3. Distribution of the electric potential produced by two keen planes held at potentials U_S and V_G. Potential is encoded by colour, increasing from blue to red, while arrows represent field lines

(see Fig. 3), where the origin of the coordinate grid is located between the two gates and lies in their plane.

The maximum electric field in the graphene plane ($y = -d_{eff}$) is achieved at $x = 0$ and equals:

$$F = \frac{V_G - U_S}{\pi\sqrt{\left(\frac{d_g}{2}\right)^2 + \left(\frac{\varepsilon_b}{\varepsilon_t} d_t\right)^2}}, \tag{14}$$

Having obtained the junction field F, we calculate the WKB transparency of the potential barrier $\mathscr{D}(p_\perp, E)$ separating the conduction and valence bands. Assuming that the potential energy depends on x - coordinate linearly, we find

$$\mathscr{D}(p_\perp, E) = \exp\left[-\frac{2}{\hbar}\int_{x_1}^{x_2} \mathrm{Im} p_\parallel(x)\,dx\right] = \exp\left\{-\frac{2}{\hbar}\int_{x_1}^{x_2} \mathrm{Im}\sqrt{p^2\left[E + eFx,\, \Delta(x)\right] - p_\perp^2}\,dx\right\}, \tag{15}$$

where $p_\parallel$ and $p_\perp$ are the components of electron quasimomentum that are parallel and perpendicular to the direction of junction field, respectively, E is the electron energy measured from the midgap in the source region, the integrals are taken between the classical turning points x_1 and x_2. Generally, the interlayer asymmetry parameters in the source (Δ_S) and in the channel (Δ_C) are different. For simplicity, we perform a linear interpolation between Δ_S and Δ_C in the junction region,

$$\Delta(x) = \Delta_S + \frac{\Delta_C - \Delta_S}{l} x = \Delta_S + \frac{\Delta_C - \Delta_S}{\varphi_C - \varphi_S} Fx, \tag{16}$$

where φ_S and φ_C are the electric potentials in the source and channel regions (obtained in the previous section), and l is the length of the transition region between them.

The tunneling current density J_{tun} can be written as

$$J_{tun} = eg \sum_{\substack{\mathbf{p} \\ v_\parallel > 0}} v_\parallel(\mathbf{p})\, \mathscr{D}(\mathbf{p}) \left[f_S(\mathbf{p}) - f_C(\mathbf{p})\right], \tag{17}$$

where $v_\parallel = \partial E / \partial p_\parallel$ is the component of electron group velocity parallel to the current direction, and f_S and f_C are the Fermi-Dirac distributions in the source and channel, respectively. It is more convenient to express all quantities in Eq. (17) via

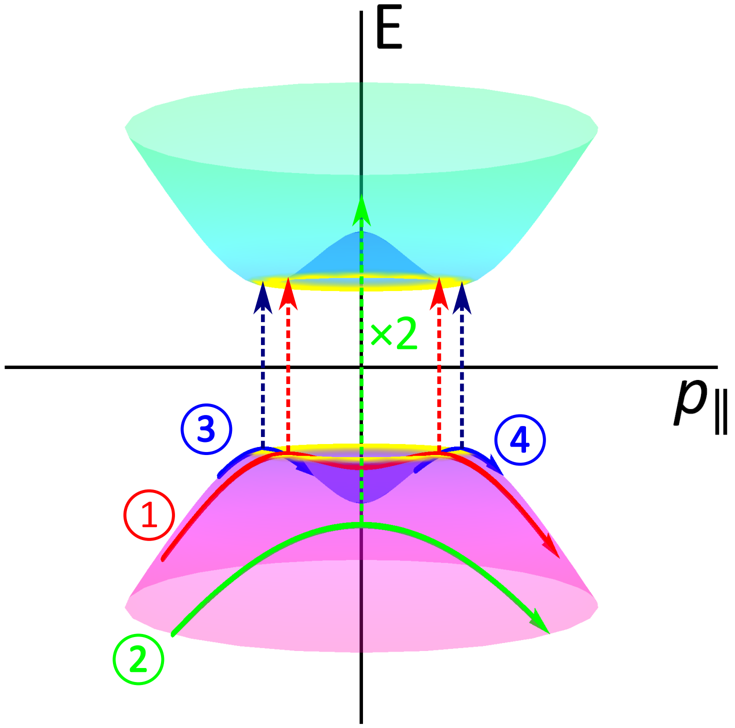

Figure 4. Illustration of interband tunneling in bilayer graphene. Valence band electrons with small transverse momentum and energy far from the band edges (1) pass through the two extrema of the dispersion law while moving in the constant electric field and may tunnel from each of these extrema. Electrons with large transverse momentum (2) pass through just one maximum. However, during their motion in the forbidden region, there are also two tunneling paths. Finally, for the energies close to the valence band edge in Eq. (18), $-\frac{\Delta}{2} < E < -\frac{\tilde{\Delta}}{2}$, there are two electron states with positive group velocity [(3) and (4)] which contribute to the current independently

the *transverse* electron momentum $p_\perp$ and its total energy E which are conserved during the tunneling:

$$J_{tun} = 2eg \int\limits_{E_c}^{E_v} [f_S(E) - f_D(E)] \frac{dE}{2\pi\hbar} \int\limits_{-p_{\max}(E)}^{+p_{\max}(E)} \mathscr{D}(p_\perp, E) \frac{dp_\perp}{2\pi\hbar}, \tag{18}$$

where $f_S(E) \equiv f(E, \mu_S)$, $f_D(E) \equiv f(E, \mu_D)$, μ_S and μ_D are the chemical potentials in source and drain regions, respectively (we assume the electrons in the conduction band of the channel region are in equilibrium with the drain contact), $p_{\max}(E)$ is the maximum transverse momentum allowed for given total electron energy E in the valence band of source and in the conduction band of the channel. More precisely, $p_{\max}(E) = \min\{p_c(E), p_v(E)\}$, where $p_c(E)$ and $p_v(E)$ are the inverse functions of the electron dispersion law $E(p)$ in the conduction band of the channel and the valence band of source, respectively.[7] Finally, we note the appearance of the factor of two before the whole expression for the tunnel current which is unique to the band structure of graphene bilayer. It appears due to the presence of two points with zero group velocity in the dispersion laws for electrons and holes, which implies that a carrier incident on a barrier attempts to pass through it twice (see the red arrow in Fig. 4).

Thermionic currents J_e and J_h carried by electrons and holes, respectively, are calculated in a similar way, except for the absence of the factor of two and barrier transparency equal to unity. The integration limits E in the expressions for the

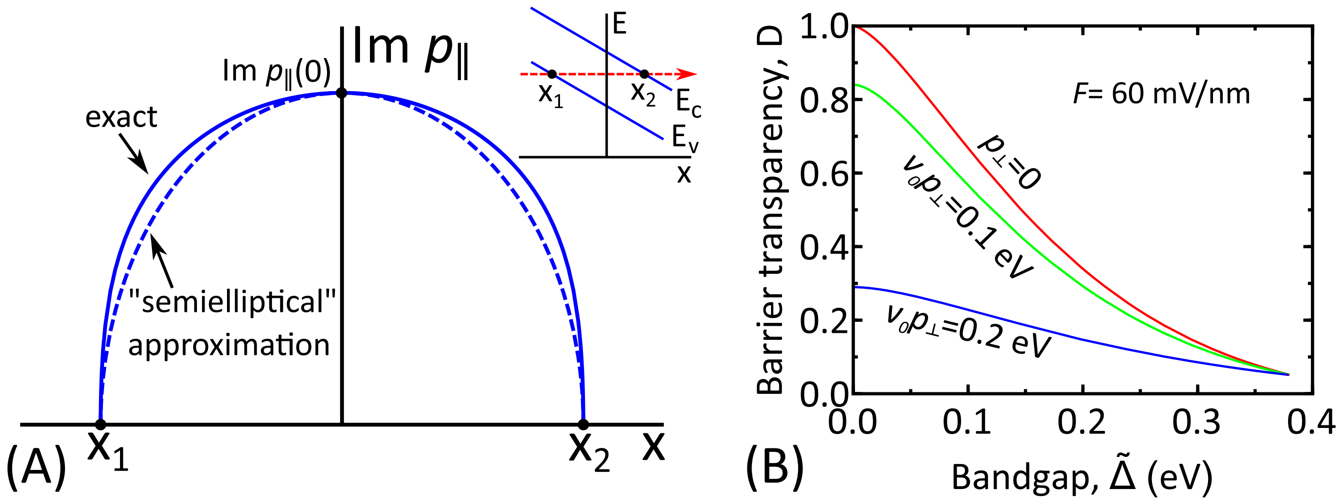

Figure 5. (A) Exact and approximate imaginary part of electron longitudinal momentum in the barrier. Electron with given energy and transverse momentum exits valence band at point x_1, tunnels through the bandgap, and enters conduction band at point x_2, as shown in the inset (B) Calculated dependencies of the quasi-classical barrier transparency on the bandgap $\tilde{\Delta}$ at different transverse momenta $p_\perp$.

thermionic current are restricted to the energies above the barriers created by the doping gates:

$$J_e = \frac{eg}{2\pi^2\hbar^2}\int\limits_{E_c^S}^{+\infty} p_{\max}(E)\left[f_S(E)-f_D(E)\right]dE, \tag{19}$$

$$J_h = \frac{eg}{2\pi^2\hbar^2}\int\limits_{-\infty}^{E_v^D} p_{\max}(E)\left[f_S(E)-f_D(E)\right]dE, \tag{20}$$

where E_c^S and E_v^D are the edges of conduction band in source region and valence band in drain region, respectively. Electrons from the "hat" (i.e. with $p < p_{min}$) do not contribute to the thermionic current because they can neither remain in the "hat" in all three regions (source, gate and drain) nor get out of it (it is possible only at band edges).

Derivation of approximate analytic expressions for tunnel and thermionic currents

Firstly, we need to obtain an analytic expression for barrier transparency. The integrand in Eq. (15) is too complicated to carry out the integration exactly. However, using the fact that the integrand has an arch-like shape (it turns to zero at x_1 and x_2 and reaches maximum somewhere between), we can approximate it with a half ellipse of the same width and height (see Fig. 5 A).

In the simplest case of constant $\Delta = \Delta_{tun}$ the maximum of the integrand is reached in the middle of the barrier and equals

$$\max \operatorname{Im} p_\|(x) = \operatorname{Im}\sqrt{p^2(0,\Delta_{tun}) - p_\perp^2} = \operatorname{Im}\sqrt{\frac{1}{v_0^2}\left(\frac{\Delta_{tun}^2}{4} + \frac{\gamma_1\Delta_{tun}}{2}i\right) - p_\perp^2}. \tag{21}$$

The main contribution to the tunnel current is from electrons with $p_\perp < p_{min}$, because for them barrier is the lowest and the shortest. Such electrons tunnel from the edge of the valence band to the edge of the conduction band, which gives the following barrier length:

$$x_2 - x_1 = \frac{\tilde{\Delta}_{tun}}{eF}. \tag{22}$$

Equations (15), (21) and (22) yield the following expression for barrier transparency in the "semielliptical" approximation:

$$\mathscr{D}(p_\perp) \approx \exp\left(-\frac{\pi\tilde{\Delta}_{tun}}{2\hbar eFv_0}\operatorname{Im}\sqrt{\frac{\Delta_{tun}^2}{4} + \frac{\gamma_1\Delta_{tun}}{2}i - v_0^2p_\perp^2}\right), \tag{23}$$

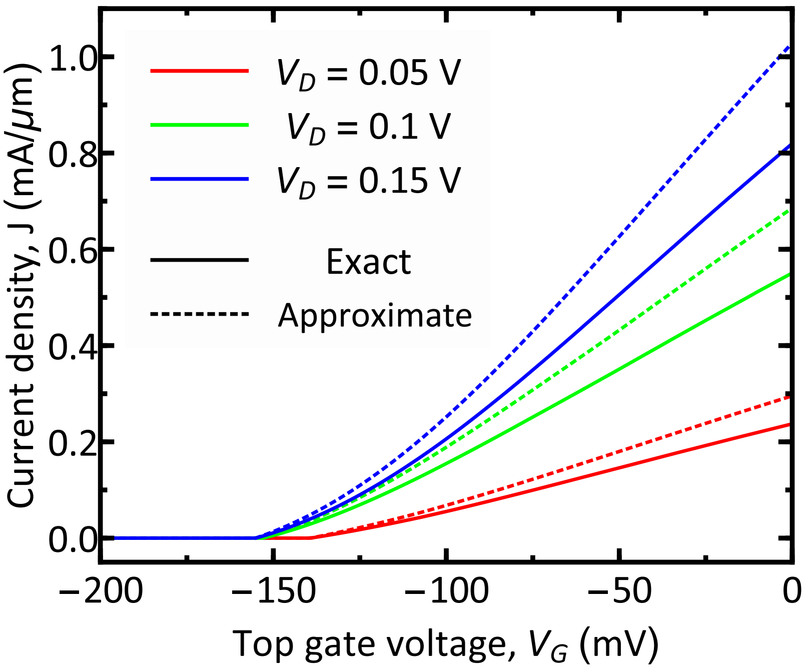

Figure 6. Comparison of gate transfer characteristics at different drain bias obtained via numerical calculations (solid lines) and via approximate analytic equations (dashed lines). In the proposed GBL TFET tunneling occurs through varying bandgap, so we chose $\Delta_{tun} = \Delta_S$ in order to partially compensate the increase in current introduced by integrating up to infinite transverse momenta

which does not depend on electron energy. The dependence of barrier transparency $\mathscr{D}$ on the bandgap $\tilde{\Delta}$ at various transverse momenta is shown in Fig. 5 B.

To be able to perform the integration with respect to $p_\perp$ in Eq. (18), we expand the exponent in Eq. (23) in a Maclaurin series up to the second order:

$$\mathscr{D}(p_\perp) \approx \mathscr{D}_0 \exp\left(-\frac{p_\perp^2}{p_0^2}\right), \tag{24}$$

where

$$\mathscr{D}_0 = \exp\left(-\frac{\pi\sqrt{\alpha-1}}{4\sqrt{2}}\frac{\Delta_{tun}\tilde{\Delta}_{tun}}{\hbar e F v_0}\right), \tag{25}$$

$$p_0 = \sqrt{\frac{2\sqrt{2}}{\pi}\frac{\alpha}{\sqrt{\alpha-1}}\frac{\Delta_{tun}}{\tilde{\Delta}_{tun}}\frac{\hbar e F}{v_0}}, \tag{26}$$

and $\alpha = \sqrt{1+\left(\frac{2\gamma_1}{\Delta_{tun}}\right)^2}$. To avoid special functions, we extend the integration region up to infinite $p_\perp$ and obtain

$$J_{tun} \approx \frac{egkT}{2\pi^{3/2}\hbar^2}\mathscr{D}_0 p_0 \ln\left[\frac{f_S(E_v)\,f_D(E_c)}{f_S(E_c)\,f_D(E_v)}\right]. \tag{27}$$

Now we move on to the calculation of the thermionic current. Near the band edges the dispersion law is approximately parabolic:

$$p_{\max}(E) \approx p_{\min} + \frac{\Delta\gamma_1}{2v_0^2 p_{\min}}\sqrt{\pm\frac{E}{\tilde{\Delta}} - \frac{1}{2}}, \tag{28}$$

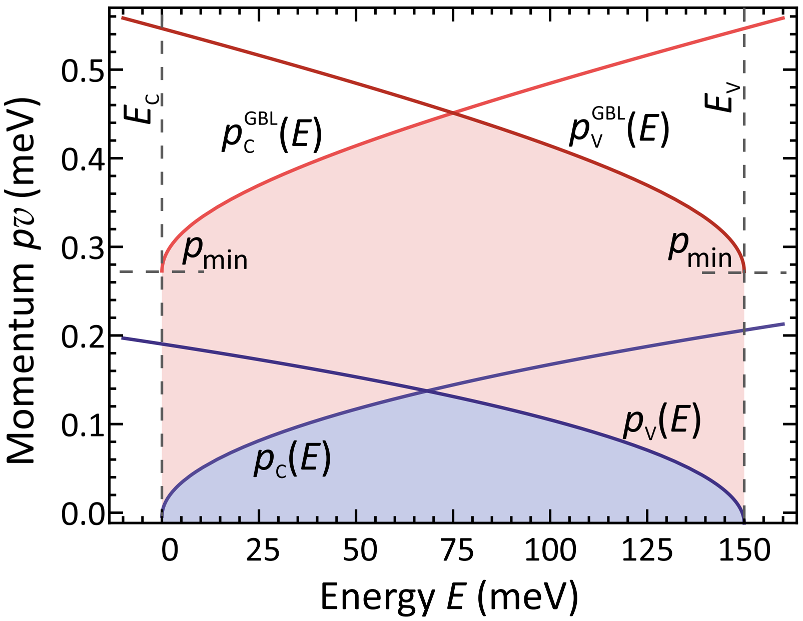

Figure 7. Energy dependencies of electron momentum in the overlapping conduction and valence bands in a voltage-biased tunnel junction. Red curves are for graphene bilayer ($\tilde{\Delta} = 0.3$ eV), blue curves are for an equivalent parabolic-band 2D semiconductor. The filled area under the curves is proportional to the interband tunnel current.

where plus sign corresponds to the conduction band and minus sign corresponds to the valence band. Taking into account that in Eq. (19) the Fermi-Dirac distribution almost coincides with the Boltzmann distibution, we obtain

$$J_e \approx \frac{egkT}{2\pi^2\hbar^2}\left(p^S_{\min} + \frac{\Delta_S\gamma_1}{4v_0^2 p^S_{\min}}\sqrt{\frac{\pi kT}{\tilde{\Delta}_S}}\right)\left[f_S\left(E^S_c\right) - f_D\left(E^S_c\right)\right], \tag{29}$$

$$J_h \approx \frac{egkT}{2\pi^2\hbar^2}\left(p^D_{\min} + \frac{\Delta_D\gamma_1}{4v_0^2 p^D_{\min}}\sqrt{\frac{\pi kT}{\tilde{\Delta}_D}}\right)\left[f_S\left(E^D_v\right) - f_D\left(E^D_v\right)\right], \tag{30}$$

where index ”S” corresponds to source, and ”D” corresponds to drain.

Comparison with TFETs based on 2d semiconductors with parabolic bands

In this section, we compare the dependencies of interband tunnel current on the band overlap in GBL and an equivalent 2D semiconductor without the ’Mexican hat’ band structure. The equivalence of the materials implies equal values of band gap and barrier transparency in the electric field, while the band structure outside the gap is different. For the sake of analytical traceability, we assume the barrier transparency $\mathscr{D}_0$ to be weakly dependent on electron energy and gate voltage. The low-energy band structure of GBL is given by Eqs. (2). The electron dispersion of a parabolic-band material is

$$E_c\left(p\right) = E_G/2 + \frac{p^2}{2m_c} \tag{31}$$

in the conduction band,

$$E_v\left(p\right) = -E_G/2 - \frac{p^2}{2m_v}, \tag{32}$$

in the valence band. Here m_c and m_v are the effective masses for in the respective bands. The interband tunneling occurs mainly between conduction and light hole bands as the barrier transparency is small for heavy carriers.

Similarly to the derivation of Eq. (2) of the main text, one can write down the current density in a parabolic-band 2d semiconductor

$$J_{\mathrm{t,par}} \approx e\frac{g_s}{h^2}\mathscr{D}_0\int\limits_{E_c}^{E_v} dE\left[f_v(E) - f_c(E)\right]\int\limits_0^{p_{\max}(E)} 2dp \approx 2e\frac{g_S}{h^2}\mathscr{D}_0\int\limits_{E_c}^{E_v} p_{\max}(E)dE, \tag{33}$$

where $p_{\max}(E)$ is the maximum transverse momentum of electron with energy E in the conduction and valence bands. The value of $p_{\max}(E)$ is the minimum of electron momenta at given energy in the conduction and valence bands:

$$p_{\max}(E) = \min\{p_c(E), p_v(E)\} = \min\left\{\sqrt{2m_c(E-E_c)}, \sqrt{2m_v(E_v-E)}\right\}. \tag{34}$$

Graphically, the current $J_{\mathrm{t,par}}$ is proportional to the area under the inverse dispersion laws in the conduction and valence bands, this region is filled with blue in Fig. 7. The integral in (33) is evaluated analytically with the result

$$J_{\mathrm{t,par}} \approx e\frac{4\sqrt{2}}{3}\frac{g_S}{h^2}\sqrt{\frac{m_c m_v}{m_c+m_v}}\mathscr{D}_0(E_v-E_c)^{3/2}. \tag{35}$$

In graphene bilayer, the tunneling current density is given by

$$J_{rmt} \approx 2e\frac{g_s g_v}{h^2}(2\mathscr{D}_0)\int\limits_{E_c}^{E_v} p_{\max}(E)dE, \tag{36}$$

where an extra factor of $g_v = 2$ accounts for the valley degeneracy, and the factor of two in front of the barrier transparency is due to the two points in electron dispersion $E_c(p)$ with zero group velocity where an incident electron attempts to tunnel from the valence band. Again, $p_{\max}(E) = \min\{p_c(E), p_v(E)\}$, where $p_{c,v}(E)$ are the dependencies of electron momentum on energy in the conduction and valence bands. Inverting the electron dispersion, one finds

$$p_c(E) = \frac{1}{v_0}\sqrt{(E-E_c)^2 + \frac{\Delta^2}{4} + \sqrt{(E-E_c)^2(\gamma_1^2+\Delta^2) - \frac{\gamma_1^2\Delta^2}{4}}}, \tag{37}$$

$$p_v(E) = \frac{1}{v_0}\sqrt{(E_v-E)^2 + \frac{\Delta^2}{4} + \sqrt{(E_v-E)^2(\gamma_1^2+\Delta^2) - \frac{\gamma_1^2\Delta^2}{4}}}. \tag{38}$$

The energy integral (36) for GBL cannot be evaluated analytically. However, the area under the dependence $p_{\max}(E)$ is naturally larger for graphene bilayer than for a typical narrow-gap semiconductor, not to mention an extra factor of four due to valley degeneracy and twofold tunneling. At small energies close to E_c, $p_c(E) \approx p_{\min} > \sqrt{2m_c(E-E_c)}$, while at large energies $p_c(E) \approx E/v_0$, which again lies above $\sqrt{2m_c(E-E_c)}$. In Fig. 5 of the main text, we numerically compare the current densities for GBL and 2d semiconductor with parabolic bands using Eqs. (36) and (35). At the operating voltage of 150 mV, the current in GBL is 15 times larger than the current in a parabolic band semiconductor, of which the factor of 2 is due to the extra valley degeneracy, the factor of 2 is due to the two-fold tunneling, and the factor of 3.5 is due to the 'Mexican hat' dispersion.

Estimate of the band tails in graphene bilayer

The abruptness of tunnel switching is limited by the tunneling from the localized states in the band tails.[8] This mechanism can be alternatively interpreted as tunneling from the band tails, produced by the random dopant-induced potential fluctuations. In this section, we evaluate the density of states in graphene bilayer due to these fluctuations. In the quasi-classical limit, the 'true' density of states $\rho(E)$ is evaluated by integrating the bare density of states shifted by eV, $\rho_0(E-eV)$, timed by the probability of voltage fluctuation of magnitude eV

$$\rho(E) = \frac{1}{\sqrt{2\pi\langle V^2\rangle}}\int\limits_{-\infty}^{+\infty}\rho_0(E-eV)\exp\left(-\frac{V^2}{2\langle V^2\rangle}\right)dV. \tag{39}$$

In the above equation, $\langle V^2\rangle$ is the root-mean square voltage fluctuation,

$$\langle V^2\rangle = \frac{1}{S}\int d\mathbf{x}V^2(\mathbf{x}) = \frac{n_i}{(2\pi)^2}\int d\mathbf{q}|V_0(\mathbf{q})|^2, \tag{40}$$

where $V_0(\mathbf{q})$ is the Fourier transform of the potential produced by a single impurity and n_i is the impurity density. To obtain a non-divergent result for fluctuations in two dimension, a finite distance from the charged impurities to the 2d plane has to be considered,[9] in this case the Fourier transform of potential is

$$V_0(\mathbf{q}) = \frac{2\pi e}{\kappa q\varepsilon(q)}e^{-qd_i}. \tag{41}$$

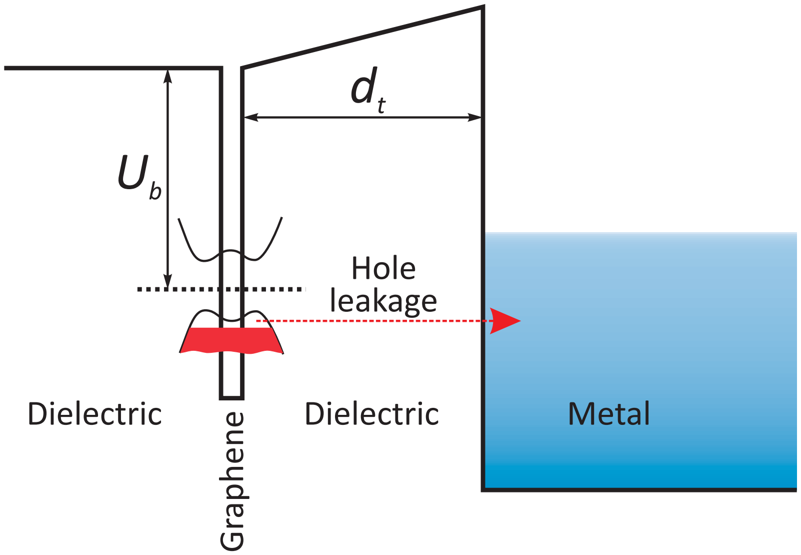

Figure 8. A model band diagram of graphene bilayer – dielectric – metal system used for the evaluation of gate leakage current. Graphene bilayer is modelled as a delta-well with potential $U(z) = -\alpha\delta(z+d_t)$, where α is chosen to provide a correct work function from graphene to the insulating material.

Above, κ is the background dielectric constant and $\varepsilon(q)$ is the dielectric function of graphene bilayer itself. For analytical traceability, the latter is taken in the Thomas-Fermi screening approximation:[10]

$$\varepsilon(q) = 1 + q_s/q, \tag{42}$$

where the screening wave vector is calculated as

$$q_s = \frac{2\pi e^2}{\kappa}\frac{1}{kT}\int_{-\infty}^{+\infty}\rho(E)f(E)[1-f(E)]dE. \tag{43}$$

The integral (40) is evaluated analytically with the result

$$\langle V^2 \rangle = 2\pi n_i \left(\frac{e}{\kappa}\right)^2 K(q_s d_i), \tag{44}$$

where $K(x) = -e^{2x}(2x+1)\mathrm{Ei}(-2x) - 1$. Equations (39) and (44) were used to calculate the density of states in the Fig. 5 of the main text numerically. The evaluation of DoS is, actually, an iterative procedure as the screening wave vector depends on the DoS itself. However, it converges quickly and the first approximation already is close to the final result.

Modeling of the gate leakage

To estimate the leakage current from graphene bilayer to the metal gate, one has to multiply the charge density in graphene by the area of the gate and by the characteristic 'tunneling frequency' ν_t. The latter can be derived from a lifetime of electron state bound to graphene bilayer in the presence of continuum of states representing the metal gate (Fig. 8).

The highest voltages are applied to the 'doping' gates, and the largest leakage is expected therein. An example of the band diagram corresponding to source doping gate is shown in Fig. 8. Graphene bilayer is modelled as a delta-well $U(z) = -\alpha\delta(z+d_t)$. The parameter $\alpha = 2\hbar\sqrt{U_b/2m^*}$ is chosen to provide a correct value of the work function from graphene bilayer to the surrounding insulator material, m^* is the electron effective mass in the insulator.

A simple wave function matching procedure for the potential profile of Fig. 8 leads to the dispersion equation for the quasi-bound states in graphene bilayer

$$(\kappa - \kappa_B)(\kappa - ik_F) = e^{-2\kappa d}\kappa_B(\kappa + ik_F), \tag{45}$$

where $\kappa_B = \sqrt{2mU_b}/\hbar$ is the decay constant of the wave function, k_F is the electron wave vector in metal (approximately equal to the Fermi wave vector), and $\kappa = \sqrt{-2mE}/\hbar$ is related to the sought-for state energy $E < 0$. The solutions of Eq. (45) are

complex, $E \approx -U_b + i\hbar\nu_t/2$, where

$$\nu_t = \frac{8U_b}{\hbar}\frac{\kappa_B k_F}{\kappa_B^2 + k_F^2}\exp\left[-2\kappa_B d\right]. \qquad (46)$$

For non-rectangular barriers, the exponent $e^{-2\kappa_B d}$ should be replaced with the quasi-classical transparency of the barrier separating graphene bilayer and the gate. The full leakage current density (per unit width of the channel) is evaluated as

$$J_g = eL_g\nu_t(n_e + n_h), \qquad (47)$$

where n_h is the density of holes induced at the source doping gate (4.5×10^{12} cm^{-2} for $U_S = -0.6$ V) and n_e is the electron density at the drain doping gate (8×10^{12} cm^{-2} for $U_D = 0.25$ V). To provide minimum tunneling frequency, we choose zirconium oxide as a gate dielectric due to its relatively large effective (tunneling) mass $m^* = 0.3m_0$ and small electron affinity $\chi_{\mathrm{ZrO_2}} = 1.6$ eV leading to a quite large value of $U_b = \chi_{\mathrm{Gr}} - \chi_{\mathrm{ZrO_2}} = 2.9$ eV.

References

1. McCann, E. & Koshino, M. The electronic properties of bilayer graphene. *Rep. Prog. Phys.* **76**, 056503 (2013).
2. Ortega, J. M. & Rheinboldt, W. C. *Iterative Solution of Nonlinear Equations in Several Variables* (Academic Press, 1970).
3. Minton, R. & Glicksman, R. Theoretical and experimental analysis of germanium tunnel diode characteristics. *Solid-State Electron.* **7**, 491–500 (1964).
4. Meyerhofer, D., Brown, G. A. & Sommers, H. S. Degenerate germanium. I. Tunnel, excess, and thermal current in tunnel diodes. *Phys. Rev.* **126**, 1329–1341 (1962).
5. Morgan, J. V. & Kane, E. O. Observation of direct tunneling in germanium. *Phys. Rev. Lett.* **3**, 466–468 (1959).
6. Ryzhii, V. & Khrenov, G. High-frequency operation of lateral hot-electron transistors. *IEEE Trans. El. Dev.* **42**, 166–171 (1995).
7. Kane, E. O. Theory of tunneling. *J. Appl. Phys.* **32**, 83–91 (1961).
8. Kane, E. O. Thomas-Fermi approach to impure semiconductor band structure. *Phys. Rev.* **131**, 79–88 (1963).
9. Adam, S., Hwang, E., Galitski, V. & Sarma, S. D. A self-consistent theory for graphene transport. *Proc. Nat. Acad. Sci.* **104**, 18392–18397 (2007).
10. Hwang, E. H. & Das Sarma, S. Screening, Kohn anomaly, Friedel oscillation, and RKKY interaction in bilayer graphene. *Phys. Rev. Lett.* **101**, 156802 (2008).
